# Economic impact of metagenomic next-generation sequencing versus traditional bacterial culture for postoperative central nervous system infections using a decision analysis mode: study protocol for a randomized controlled trial

**DOI:** 10.1128/msystems.00581-23

**Published:** 2023-11-08

**Authors:** Ying Tian, Ran Gao, Yumei Wang, Yimin Zhou, Shanshan Xu, Yuqing Duan, Wenyi Lv, Shuya Wang, Mengxue Hou, Yuqing Chen, Fangqiang Li, Wei Gao, Linlin Zhang, Jian-Xin Zhou

**Affiliations:** 1 Department of Critical Care Medicine, Beijing Tiantan Hospital, Capital Medical University, Beijing, China; 2 Department of Clinical Diagnosis, Laboratory of Beijing Tiantan Hospital, Capital Medical University, Beijing, China; 3 Department of Microbiology and Immunology, Peter Doherty Institute, University of Melbourne, Melbourne, Victoria, Australia; 4 Beijing Engineering Research Center of Digital Healthcare for Neurological Diseases, Beijing, China; 5 Beijing Shijitan Hospital, Capital Medical University, Beijing, China; University of California, San Francisco, California, USA

**Keywords:** central nervous system infections, metagenomic next-generation sequencing, cerebrospinal fluid culture, health economics, diagnosis

## Abstract

Central nervous system infections (CNSIs) are common complications after neurosurgery with a poor prognosis. The traditional microbiological culture methodology has a low detection rate and time consuming. Metagenomic next-generation sequencing (mNGS) has demonstrated the advantages of being faster, more accurate, and more comprehensive in clinical microbiology. Previous studies had suggested that mNGS had a high sensitivity in the diagnosis of CNSIs. Whether the application of mNGS has health economic value in clinical applications remains to be studied. We designed a prospective, single-center, superiority randomized controlled trial to compare the cost-effectiveness of mNGS with traditional methods for diagnosing CNSIs using a decision tree model. A total of 204 patients will be enrolled and randomly assigned to either the mNGS group or the traditional method group. The two groups of patients entered different decision points according to different clinical manifestations and examination results. They will be then given treatment decisions by a panel of specialists at the corresponding decision point. The primary outcome is the incremental cost-effectiveness ratio, which is the increased cost for every 1% increase in recovery rate. The secondary outcomes are a comparison of time cost, detection cost, and costs associated with antibiotics treatment between the two groups.

**IMPORTANCE:**

Diagnosing and treating postoperative central nervous system infections (PCNSIs) remains challenging due to the low detection rate and time-consuming nature of traditional methods for identifying microorganisms in cerebrospinal fluid. Metagenomic next-generation sequencing (mNGS) technology provides a rapid and comprehensive understanding of microbial composition in PCNSIs by swiftly sequencing and analyzing the microbial genome. The current study aimed to assess the economic impact of using mNGS versus traditional bacterial culture-directed PCNSIs diagnosis and therapy in post-neurosurgical patients from Beijing Tiantan Hospital. mNGS is a relatively expensive test item, and whether it has the corresponding health-economic significance in the clinical application of diagnosing intracranial infection has not been studied clearly. Therefore, the investigators hope to explore the clinical application value of mNGS detection in PCNSIs after neurosurgery.

## INTRODUCTION

Central nervous system infections (CNSIs) are severe complications after neurosurgery that can lead to a poor prognosis (1, 2). The incidence of postoperative CNSIs (PCNSIs) ranges from 2.8% to 14% (3), and there are differences between different regions. The incidence rate in developed countries is lower than that in developing countries. The most common manifestations of PCNSIs include meningitis, ventriculitis, subdural abscesses, epidural abscesses, and brain abscesses. Studies have shown that the most common pathogens of PCNSIs are *Staphylococcus aureus*, and *coagulase-negative Staphylococcus*, followed by gram-negative bacteria (4). In addition, culture as the gold standard is time-consuming and susceptible to the use of antibiotics. PCNSIs are associated with significantly increased treatment costs, prolonged hospitalization time, psychological trauma, and delayed postoperative adjuvant treatment. These factors can place a substantial economic and psychological burden on society and patients’ families (5).

Given the gravity of PCNSIs, the selection of appropriate antibiotic treatment is crucial but challenging and should be guided by pathogen identification and antimicrobial susceptibility. Therefore, rapid and accurate pathogen characterization is imperative for PCNSIs. Compared to traditional pathogenic microbial detection methods, metagenomic next-generation sequencing (mNGS) offers several advantages, including enhanced speed, accuracy, and comprehensiveness (6). mNGS is increasingly utilized in the diagnosis of CNSIs, respiratory infections, blood infections, and other acute, critical, and complex infections (7, 8). Research indicates that mNGS demonstrates higher positivity rates than culture methods and is less affected by antibiotic use, providing more precise information on the patient’s infection status (9–11). Additionally, mNGS enables the detection of various pathogen types, offering more effective treatment guidance. Furthermore, the fast turnaround time (TAT) of mNGS contributes to significantly shorten the disease course and improve the prognosis for infected patients (12).

While most published studies have focused on evaluating the clinical diagnostic value of mNGS, concerns regarding its comprehensive clinical utility persist, mainly due to cost constraints. The overall expenses associated with mNGS detection reagents and labor exceed those of traditional detection methods (13). Currently, no health economics research has been conducted on the application of mNGS for diagnosing CNSIs following neurosurgery. Therefore, prospective clinical trials are necessary to assess the cost-effectiveness of mNGS as a relatively expensive novel detection method.

In summary, this study aims to conduct a health economics investigation of mNGS for diagnosing PCNSIs and evaluate whether this relatively costly technology can facilitate early pathogen identification, reduce the duration of anti-infective treatment, lower overall medical expenses, and enhance patient recovery rates. Furthermore, this research provides theoretical guidance for clinical and public health departments to make scientifically informed, cost-effective decisions, optimize medical resource allocation, and improve the societal and economic benefits of etiological diagnosis.

## MATERIALS AND METHODS

### Study design

**Fig 1:**
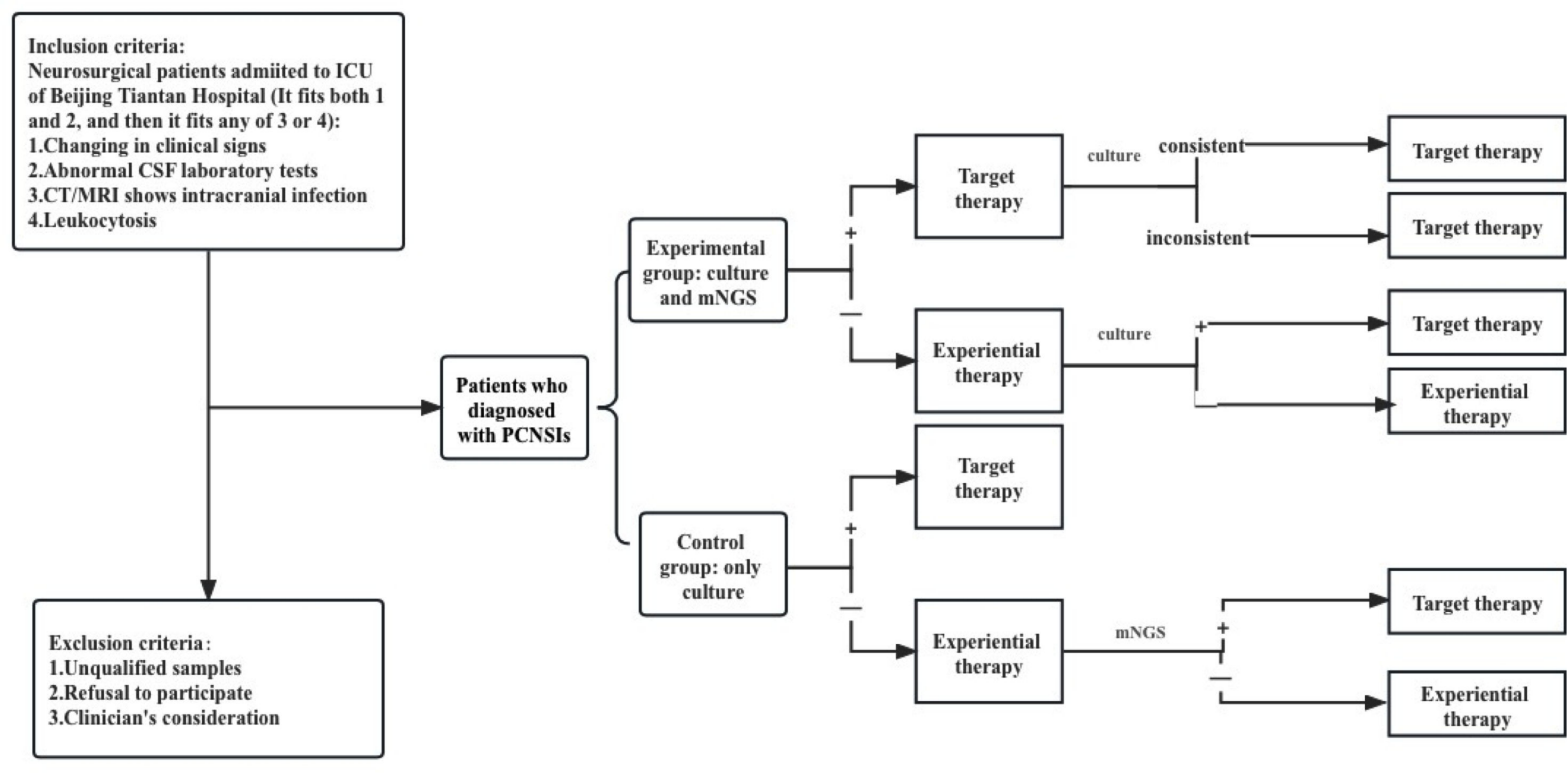
Flow chart: patient inclusion criteria, exclusion criteria, and decision-making process.

This is a single center, superiority randomized controlled trial enrolling patients with CNSIs after neurosurgery. The enrolled patients will be divided into two parallel groups in a 1:1 ratio: those diagnosed by CSF pathogen culture and metagenomic sequencing (experimental group) and those diagnosed using pathogen culture alone (control group). A detailed schematic of the study is shown in Fig. 1. For inclusion in patients with a comprehensive clinical diagnosis of CNSIs after neurosurgery, the inclusion criteria are shown in Table 1. Exclusion criteria are patients with unqualified samples, patients, and their families who refused to sign the informed consent, or the clinician who considered the case unsuitable for inclusion in the study. Subjects may discontinue the study at any time for safety or personal reasons. We set up a group of experts composed of two clinical experts, microbial experts, bioinformatic analysis experts, and experimental technicians.

**TABLE 1:** Clinical comprehensive diagnostic criteria

It fits both 1 and 2, and then it fits any of 3 or 4	
1. Clinical signs (consistent with any point)	Changes in consciousness and mental state: new delirium, irritability, lethargy, and coma. Increased intracranial pressure: headache, vomiting, and papilledema. Meningeal stimulation signs: cervical stiffness, Kernig sign (+), and Brudzinski sign (+). Symptoms of systemic infection: T > 38°C or T < 36°C, increased heart rate and respiration.
2. Cerebrospinal fluid examination results	Intracranial pressure > 200 mmH_2_O. The character of cerebrospinal fluid is cloudy, yellow, or purulent. Cerebrospinal fluid leukocyte > 1,000/mm^3^ and polynuclear leukocyte > 70%. Glucose < 2.2 mmol/L and cerebrospinal fluid glucose content/serum glucose content ≤ 0.4.
3. Clinical imaging examination	Computed tomography/magnetic resonance imaging: diffuse intracerebral edema, dural thickening and enhancement, or dilation of the ventricular system, or even typical annular enhanced space occupying.
4. Blood routine	Leukocyte > 10*10^9^ /L, neutrophil ratio more than 80%.

### Protocols for the identification of the bacterial culture

The clinically collected CSF is added to the blood culture bottle and put into the automatic bacterial culture instrument as soon as possible for continuous culture for 5 days. The blood culture vial with positive alarm is inoculated into the blood plate and cultured in the automatic bacterial culture incubator for 24 hours. The pure culture colonies are identified by mass spectrometer and VITEK 2-compact. The quality control strains are *Escherichia coli* (ATCC25922), *Pseudomonas aeruginosa* (ATCC27853), and *S. aureus* (ATCC25923), and the quality control strains were from the clinical laboratory center of the Ministry of Health.

### Protocols for the identification of mNGS

In the mNGS wet assay, a negative control (sterile deionized water) and a positive control (known amount of synthetic fragments) are established for each batch. Reads and reads per million (RPM) are calculated for each detected microorganism. For detected microorganisms, including bacteria (excluding *Mycobacterium*), fungi (excluding *Cryptococcus*), and parasites, when they are in the top 10 of their class of microbial species (or genus) in terms of coverage and are not present in the negative control (“no template” control, NTC), or when the RPM ratio between sample and NTC (RPMsample/RPMNTC) > 10 and RPMNTC ≠ 0, the result of mNGS is positive. For viruses, *Mycobacterium* and *Cryptococcus*, mNGS results are considered positive when at least one unique read is mapped to the species level and was not present in the NTC or RPMsample/RPMNTC > 5 and RPMNTC ≠ 0.

### Informed consent

Once the patient is confirmed to be eligible for inclusion, informed consent will be signed by the doctor and the patient or authorized representative. Each relevant aspect of the project will be described, and written informed consent of the patient will be obtained. If the patient is unable to consent, written informed consent will be obtained from his or her authorized representative. The doctor will assure the patient or their authorized representative that they are free to withhold consent without consequences, and they can withdraw consent at any time without compromising treatment.

### Patient and clinical evaluation of enrolled patients

Patients who met the inclusion criteria were enrolled in the study after signing informed consent. Basic information of enrolled patients was collected, such as sex, age, primary diagnosis, medical history, and operation time. The date of comprehensive clinical diagnosis, CSF collection, mNGS detection, pathogen culture, and reporting date should be recorded. The patient’s clinical signs, such as changes in mental state and body temperature, were collected. Laboratory test results were collected, including routine blood, procalcitonin, routine CSF, CSF biochemistry, and pathogen culture results. For patients with suspected CNSIs, the neurosurgeon will evaluate the suitability for transport imaging based on the patient’s vital signs. Patients with stable vital signs will be examined with computed tomography and magnetic resonance imaging.

### Sample size calculation

We calculated the sample size by “Superiority by a Margin Tests for Two Survival Curves Using Cox’s Proportional Hazards Model” in PASS15. The experimental group was set as 1:1 for the control group, HR: The mortality ratio at the time of discharge. According to our previous clinical data, HR was estimated as the ratio of the mortality rate of the experimental group (P2) to that of the control group (P1), which was 0.05/0.2 = 0.25. HRsu is the largest that HR can be that still concludes clinical superiority (0 < HR < HRsu < 1), which was set as 0.8. The sample size of the experimental group was calculated as 102 cases, the control group as 102 cases, and a total of 204 patients were expected to be included. With an expected 10% case-shedding probability, 226 patients were eventually included.

### Adverse event management

In this study, an additional 2 mL of CSF will be drawn during routine clinical examinations in the experimental group of patients. However, the process of collecting additional cerebrospinal fluid (CSF), medical history, and other information can cause psychological discomfort to participants. There may also be risks. For example, some people may experience mild dizziness, headache, and pain at the puncture site, and in rare cases, an infection may develop at the needle site.

To further assess and avoid adverse events, we will record these events and have them managed by a doctor. If the patient develops psychological discomfort during history collection, the investigator will provide timely reassurance, and the event will be recorded and evaluated by a designated person. Participants with mild headaches and back pain after a lumbar puncture could receive fluids. The patient will receive appropriate treatment or compensation in the event of accidental injury or loss associated with this study. All adverse events in this clinical study will be recorded in detail. Any serious adverse events will be handled and documented by special personnel and reported to the ethics committee and relevant departments promptly.

### Model overview

From a patient perspective, we developed a decision analysis model in Microsoft Excel for the precise pathogenic diagnosis of PCNSIs in neurosurgery. Two detection strategies were considered in the model, mainly metagenomic detection and pathogen culture. This study is expected to include patients with a clinical diagnosis of CNSI after neurosurgery admitted to the intensive care unit of Beijing Tiantan Hospital. Patients were randomly divided into experimental and control groups (it should be noted that patients in both groups were clinically diagnosed with CNSI, and patients were given empiric therapy immediately without waiting for mNGS and culture results). We use blocks to randomly divide groups. According to the time when the subjects entered the study, the block size was set to 8 (i.e., each block group contained eight subjects), and 226 subjects were divided into 29 block groups. Simple randomization was used within each block to assign subjects to the experimental or control groups.

The experimental group was sent for CSF mNGS and pathogen culture simultaneously, and the mNGS results were usually earlier than the pathogen culture results. Therefore, the experimental group first adjusted or continued the current medication regimen according to the mNGS reporting pathogen and the expert team’s opinion. Subsequently, if the CSF pathogen culture results in the experimental group were consistent with the mNGS results, the current treatment plan of the patient was continued, and if the results were inconsistent with the mNGS results, the expert team discussed and adjusted the treatment plan. When no causative organism is detected in mNGS, empiric treatment is continued, and treatment is adjusted pending the pathogen culture results.

After a clinical diagnosis of CNSI, the control group was treated empirically based on only CSF for pathogen culture, without mNGS detection, and the treatment plan was adjusted according to the pathogen culture results. If the patient’s culture was negative and empiric therapy did not improve, CSF was retained for mNGS testing. Treatment is adjusted based on mNGS results and expert team evaluation.

### Decision tree

The model structure is shown in Fig. 1. After neurosurgery, patients enter the model when clinically diagnosed with CNSI of unknown etiology. The CSF of the control group was cultured, and the experimental group was tested by mNGS.

### Model inputs

#### Epidemiology and population inputs

Patients included in the study needed to have adequate tissue samples collected. The patient has Chinese nationality, records the place of household registration, medical insurance reimbursement ratio, statistics on the median age of the patient, the primary disease type (tumor, trauma, vascular disease, etc.) and its proportion, mNGS detection, and pathogen culture detect pathogen species (bacteria, viruses, fungi, etc.).

#### Testing cost

Cost estimates are mainly direct medical expenses, which are obtained from internal hospital data. Due to the different instruments and equipment required by the two detection methods and the different quantities tested during the depreciation period, it is not easy to accurately calculate the cost of the instruments. Therefore, the cost of testing mainly includes the cost of CSF pathogenic microbial culture, the cost of CSF mNGS detection (patients mainly detect DNA, and RNA is detected when RNA virus is suspected), and sample collection costs.

#### Time inputs

Patients in the included studies had a median time to the culture of CSF pathogens and a median time to mNGS detection, length of stay in the intensive care unit, length of anti-infective treatment, and total length of hospitalization.

#### Treatment and testing inputs

Medical costs include Western medicines. Laboratory, imaging, and clinical diagnostic program fees. One-time medical materials for routine examination, treatment, and surgery. General medical services and treatment operation fees. Antimicrobial fees; nursing fees. Fees for proprietary Chinese medicines and herbal medicines, etc. These directly related to infection include antimicrobial costs and culture, mNGS, and sample collection costs.

### Model outputs

#### Primary outcome

Incremental cost-effectiveness ratio (ICER): This measures the increased cost for each unit of mortality reduction or increase in cure rate. ΔC: incremental cost, i.e., total cost difference between the two groups during hospitalization; ΔE: incremental effect, i.e., the difference in a cure rate of CNSI after neurosurgery between the two groups. ICER = ΔC/ΔE.

#### Secondary outcome

Cost comparison: We conducted a comparison between the mNGS group (experimental group) and the etiology culture group (control group) to determine the disparity in total costs. Various cost components were taken into account, including time-related expenses (such as the duration spent on mNGS and culture testing, anti-infective treatment, total hospital stay, and intensive care unit (ICU) stay), detection costs (mNGS and culture), and antibiotics expenses.

Efficacy comparison: The cure and mortality rates of CNSI in the experimental and control groups were compared.

### Statistical analysis

Analysis was conducted at the level of each patient’s hospitalization in a per-protocol analysis. Measurement data are expressed as the mean and SD or median (interquartile range), and data that conform to the normal distribution were statistically analyzed using the independent sample *t*-test. Data that did not conform to the normal distribution were tested using the Wilcoxon rank sum test. Categorical variables are expressed as counts and percentages and were analyzed with χ^2^ tests.

## RESULTS AND DISCUSSION

CNSIs are characterized by substantial morbidity, fatality rates, and significant costs imposed on the healthcare system (14). In recent years, there has been an increasing prevalence of *Acinetobacter baumannii* and *Klebsiella pneumoniae* in hospital-acquired infections (15). Bacterial infections affecting the central nervous system represent a medical emergency, necessitating prompt diagnosis and immediate treatment. Despite optimal therapeutic interventions, many patients remain at risk of developing major systemic and neurological complications, resulting in high mortality rates and severe disability for survivors (16). Empirical treatment strategies rely on regional patterns of antibiotic resistance among common pathogens. In cases of subdural and brain abscesses, neurosurgical intervention is required to drain the infection and facilitate prolonged antibiotic therapy (17).

In clinical practice, pathogen culture and mNGS technology are commonly employed for detecting the etiology of CNSIs following neurosurgery (12). Previous reports have indicated that mNGS offers advantages over culture-based methods in diagnosing CNSIs (18). However, there is a dearth of systematic studies evaluating the clinical efficacy of mNGS, including prognosis and treatment outcomes (19). While rapid advancements in diagnostic methods have enabled clinicians to swiftly and accurately establish definitive diagnoses, the associated costs can be substantial. Nevertheless, there is limited evidence on the comparative economic implications of employing these two different diagnostic strategies for patients with CNSIs. Hence, our objective is to develop an economic model to assess the clinical benefits of different testing approaches.

In designing this study, we considered that although the cost of mNGS detection is higher than that of pathogen culture, its shorter turnaround time for results facilitates early initiation of targeted treatment (20–22), potentially leading to significant reductions in medical costs and improved patient prognosis, thereby combining cost-effectiveness. Nathan A. Pennell et al. suggested that “the use of second-generation sequencing may expand the number of patients undergoing testing for common and less common genetic mutations, providing physicians and patients with more information to make informed and timely treatment decisions. This can help improve outcomes and extend survival” (23).

Haibing Liu’s study suggests that the false positive rate of mNGS in diagnosing lower respiratory tract infections is higher compared to traditional microbial detection methods (24). This is attributed to the colonization of bacteria, viruses, fungi, and other microorganisms in the respiratory system, which mNGS can capture as free nucleic acids. Therefore, when interpreting mNGS results from respiratory tract samples, comprehensive clinical diagnosis should be considered (20, 25, 26). CSF is a sterile body fluid in normal conditions. Therefore, the false positive issue can be preventable by performing strict aseptic techniques. In our previous studies, the false positive rate of bacterial detection using mNGS in CSF samples was only 1.52%. Conversely, due to the relatively low pathogen load in CSF samples during CNSIs, mNGS has been challenged in capturing such a low load of pathogenic microorganisms, leading to an increased possibility of a false negative rate (27). This introduces uncertainty in the clinical health economics of CSF mNGS and pathogen culture.

The present study is the first study that focuses on the economic impact of utilizing mNGS compared to traditional culture-guided CNSIs diagnosis and treatment of post-neurosurgery patients. We designed a decision tree model. Both groups of patients will be treated at the corresponding decision point by a team of specialists. The primary outcome is the ICER. mNGS is a relatively expensive test, so our primary outcome is the increased cost for every one percent increase in the cure rate. The secondary outcomes are a comparison of time cost, detection cost, and costs associated with antibiotics treatment between the two groups. We investigated the cost and effectiveness analysis of intracranial infections in patients during hospitalization, and medical costs during hospitalization were available from the electronic medical record system. To enhance the medical care provided to patients with PCNSIs, we set up an expert team consisting of clinicians, microbiological experts, bioinformatics experts, and experimental personnel. The expert team assessed the state of consciousness, vital signs, and clinical signs of enrolled patients daily, regularly assessed relevant laboratory examinations and imaging examinations, and adjusted the treatment plan based on the obtained results. Nevertheless, it is important to acknowledge the limitations of this study. Primarily, it should be noted that this research is limited to a single-center setting, focusing on the evaluation of the health economics of mNGS specifically in critically ill patients with CNSIs. Therefore, the findings may not be directly applicable to other healthcare settings or patient populations. Second, the study exclusively compares mNGS with the traditional culture method for detection. However, there are other detection methods available, such as multiplex PCR and target panel, which warrant further investigation in future studies. These alternative methods may offer valuable insights and contribute to a more comprehensive understanding of their effectiveness and economic impact in the diagnosis and treatment of CNSIs. Acknowledging these limitations, future research should aim to expand the scope of the investigation to encompass multiple healthcare centers and consider a broader range of detection methods, thus providing a more comprehensive analysis of the health economics of various approaches in managing CNSIs.
